# Role of Maternal Infections and Inflammatory Responses on Craniofacial Development

**DOI:** 10.3389/froh.2021.735634

**Published:** 2021-09-06

**Authors:** Anjali Y. Bhagirath, Manoj Reddy Medapati, Vivianne Cruz de Jesus, Sneha Yadav, Martha Hinton, Shyamala Dakshinamurti, Devi Atukorallaya

**Affiliations:** ^1^Department of Pediatrics and Physiology, University of Manitoba, Winnipeg, MB, Canada; ^2^Biology of Breathing, Children's Hospital Research Institute of Manitoba (CHRIM), Winnipeg, MB, Canada; ^3^Department of Oral Biology, Dr. Gerald Niznick College of Dentistry, University of Manitoba, Winnipeg, MB, Canada; ^4^Mahatma Gandhi Institute of Medical Sciences, Wardha, India

**Keywords:** pregnancy, maternal inflammation, infections, fetal development, craniofacial development

## Abstract

Pregnancy is a tightly regulated immunological state. Mild environmental perturbations can affect the developing fetus significantly. Infections can elicit severe immunological cascades in the mother's body as well as the developing fetus. Maternal infections and resulting inflammatory responses can mediate epigenetic changes in the fetal genome, depending on the developmental stage. The craniofacial development begins at the early stages of embryogenesis. In this review, we will discuss the immunology of pregnancy and its responsive mechanisms on maternal infections. Further, we will also discuss the epigenetic effects of pathogens, their metabolites and resulting inflammatory responses on the fetus with a special focus on craniofacial development. Understanding the pathophysiological mechanisms of infections and dysregulated inflammatory responses during prenatal development could provide better insights into the origins of craniofacial birth defects.

## Introduction: Infections in Pregnancy and The Developing Fetus

### Pregnancy: An Immunological Balancing Act

Pregnancy has been described quite aptly as an immunological balancing act consisting of both activation and regulation. Sir Peter Medawar described the fetus as a semi-allograft that is somehow not rejected by the maternal immune system [1, 2]. Medawar proposed three possible mechanisms for the tolerance of the fetal semi-allograft: an energy or tolerance by the maternal immune system, a physical barrier such as the placenta, and, suppression of the fetal allo-antigens. Studies that followed also showed that while the embryo indeed exhibits paternal major histocompatibility complex (MHC) antigens [3, 4]. These could incite a response if recognized by the maternal immune system, a cooperative mechanism has somehow evolved which results in a successful pregnancy. It is in fact a combination of signals and responses to and from the placenta that modulates the maternal immune system.

The paternal seminal fluid contains several antigens and immunomodulatory molecules such as class Ia, Ib, and class II MHCs [5]. Murine uterine dendritic cells have been shown to cross-present the paternal antigens to activate the maternal T-cells and facilitate immune tolerance toward them [6]. Post-fertilization, the blastocyst “hatches” from the zona pellucida that surrounds it just prior to implantation. Studies on mouse embryos have shown that the embryos express MHC class I proteins [3, 4]. If the zona pellucida is removed prematurely, the maternal cytotoxic T-cells can recognize and kill these embryos [7]. This suggests a protective role for the zona pellucida toward the embryo. It has now been shown that a response by the uterine innate immune system is in fact important for a successful implantation of the embryo [8, 9]. Studies on implantation failures have found that pre-existing microbial invasion or colonization of the endometrium (as seen in sexually transmitted diseases or vaginitis) can result in failed implantations after *in vitro*-fertilization as well as spontaneous abortions [10].

Post-implantation, the primary site for materno-fetal contact is at the interface between the maternal decidua and the extravillous trophoblasts of the blastocyst. Despite being genetically distinct, somehow there is a lack of maternal antigenic stimulation toward the invading blastocyst [11, 12]. The major component of the maternal decidua is the decidual stromal cells which influence several immunologic activities. Tolerance is mediated by cytokines and the MHCs from the circulating leucocytes. This helps the mother “understand” the fetal signals and not perceive them as a threat. The interface between these two entities (the maternal decidua and the extravillous trophoblasts), shapes the immunologic relations through the course of the pregnancy.

In the first trimester, the fetal extravillous trophoblasts and the maternal decidual cells exhibit unique human leukocyte antigen (HLA) and chemokine receptor profiles [13–15]. The HLAs expressed by the fetal membranes have been found to be tolerogenic rather than immunogenic [16, 17]. Fetal trophoblasts express one class Ia MHC and three class Ib molecules. However, they do not express the class Ia antigens, HLA-A and HLA-B which are responsible for allograft rejection in humans [18, 19]. Thus, most immunomodulation is proposed to occur via regulation of innate immune responses and natural killer (NK) cells. The maternal decidua was believed to contains regulatory T-cells, macrophages and the NK cells but no B lymphocytes [20]. Benner et al. recently reported that decidual B-cells not only exist but also secrete anti-inflammatory cytokines such as IL-10 [21]. The fetal extravillous trophoblasts also express chemokine receptors such as CX3CL1, CCL14, and CCL4 [22]. The expression of chemokine receptors is suggested to facilitate the migration of the maternal leucocytes and encourages further invasion of the trophoblast [22].

The first trimester, specifically the weeks 3 through 10, is the most crucial period in craniofacial development. The term craniofacial structure is collectively used to describe the structures in the head and neck region. While craniofacial development is initiated in the first trimester of pregnancy, it continues well unto puberty. Any perturbations in the first trimester can have a deterministic effect on the normal development of these structures.

Craniofacial structures are derived from branchial arches and most of the mesenchymal structures in the head and neck are derived from the neural crest cells (NCCs). Neural crest cells after induction, undergo an epithelium-mesenchymal transition before migrating to specific locations and differentiating into several cell types. These transient NCCs are highly sensitive to environmental signals and their migration is guided by molecular signals from their non-neural crest tissue environments. Syndromic disorders arising because of defective NCC development and regulation are termed neurocristopathies. NCCs are characterized by environmentally responsive surface markers including integrin-α4, CD-49d, LPAM-1, Notch 1 and 2 and intracellular markers such as Sonic hedgehog (Shh), Sox, Snail, Slug, Vimentin etc. A discussion on specific signaling pathways and craniofacial development is outside the scope of this review as we have chosen to focus on environmental influences. NCCs are very sensitive to extrinsic factors such as ethanol exposure [23], excessive environmental glucose [24], temperature fluctuations [25], nutritional deficiencies [26] and infections [27]. Immunological investigations into NCCs identified that NCCs exhibit lower expression of HLA class I but no expression of HLA class II suggesting poor immunogenicity [28]. iPSC derived NCCs are shown to exhibit immunosuppressive properties by inhibiting T-cell proliferation and decrease in inflammatory cytokines [28].

Using live cell imaging in zebrafish, Zhu et al., demonstrated that NCCs are capable of phagocytic functions in early embryonic development [29] suggesting diverse functional roles.

**Figure 1:**
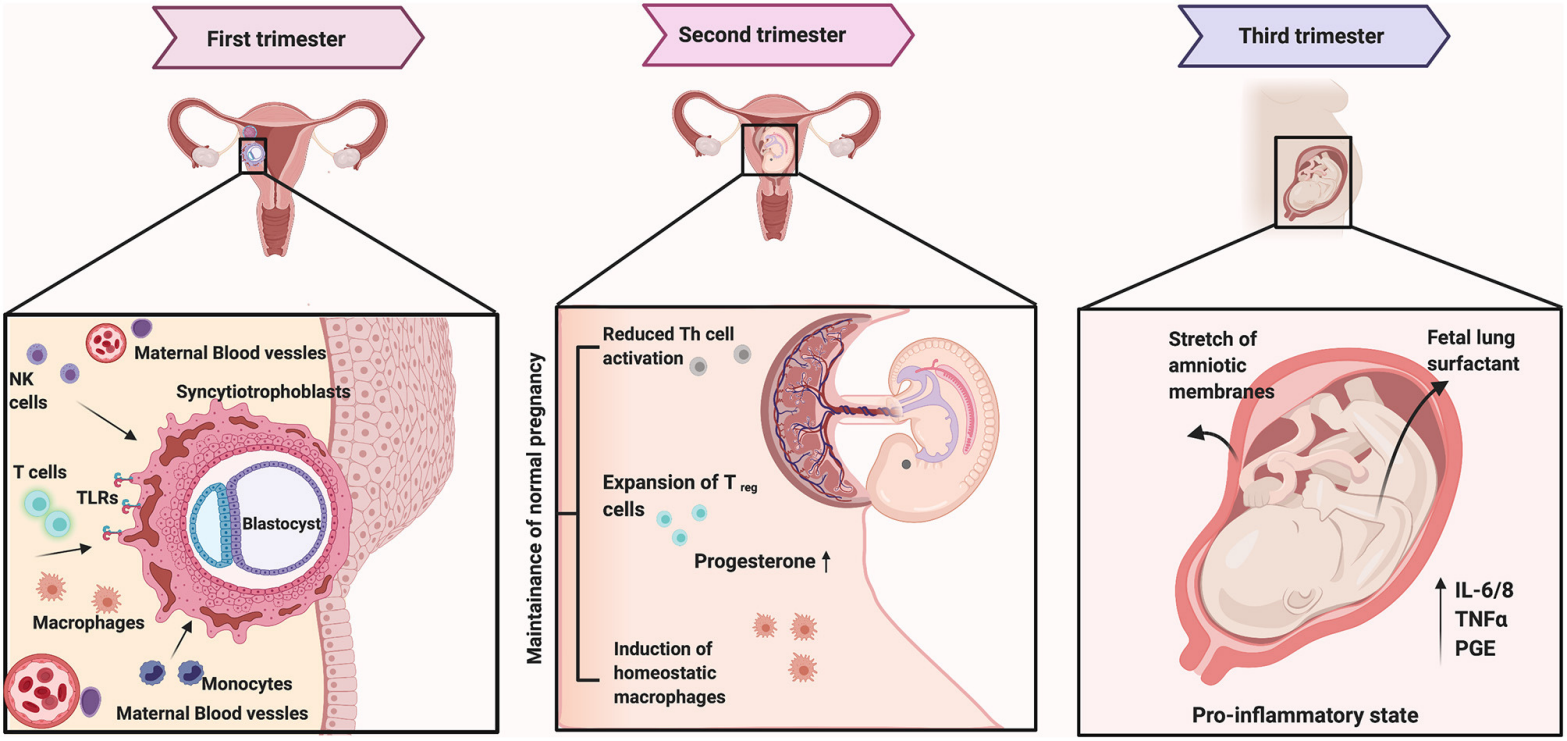
The first trimester in pregnancy is marked by activated inflammation beginning with the adherence of the blastocyst to the uterine wall. The invading trophoblasts from the blastocyst secrete chemokines to recruit maternal innate (monocytes, macrophages, and natural killer cells) and adaptive immune cells [including a restricted subset of CD4+ and CD8+ T cells and regulatory T cells (T_reg_)]. Simultaneously, there is the proliferation of resident tissue leukocytes, particularly decidual natural killer (dNK) cells and decidual dendritic cells (dDCs). Trophoblasts impart an immature phenotype to local dDCs that encourages differentiation of T_regs_ and a tolerogenic Th2-polarized environment with high levels of classically anti-inflammatory cytokines, such as IL-10. The second trimester or mid pregnancy is marked with immune senescence with increased progesterone and T_reg_ dominant response. As the pregnancy approaches term, the local indicators of fetal maturity trigger the maternal immune system to undergo a shift toward a pro-inflammatory state again with its peak at term. Further, the stretch of amniotic membranes as well as the fetal lung surfactant have been shown to activate inflammatory responses to facilitate parturition. Figure created using the BioRender software.

The first trimester (Figure 1) is characterized by regulated immune activation within the uterine environment, an idea that was established in a mouse model in 1992 [30]. The second trimester, however, is marked by immune quiescence while parturition is again an event marked by activated inflammation. Over the course of a human pregnancy, the concentration of hormones such as estrogen (estradiol and estriol) and progesterone increase with the highest levels observed during the third trimester. Hormonal changes during pregnancy can alter immune responses to increase susceptibility to infections [31, 32]. The endometrium also gets enriched with macrophages and regulatory T cells (T_reg_ cells). T_regs_ comprise of both ${\text{CD}}_{4}^{+}$ and the ${\text{CD}}_{8}^{+}$ cells. Studies have shown that the concentration of T_regs_ increases in the first trimester, peaks in the second, and decreases in the third [33–35]. Reduced number of T_regs_ are associated with recurrent pregnancy loss as well as pre-eclampsia [34, 36, 37]. In a healthy pregnancy, while the levels of circulating helper T-cells type 1 and 2 (Th1 and Th2) cells remains unchanged until the third trimester, the levels of NK cells and natural killer T-cells (NKT cells) increases [38–40]. The NK and the NKT cells respond to cytokines secreted by the dendritic cells upon activation by external stressors such as pathogens or their molecules. The cytotoxic effects of the NK cells are associated with recurrent pregnancy loss. This effect is postulated to be associated with an alteration in subset of NK cells rather than overall change in abundance [41, 42].

Elevated progesterone has a direct effect on the levels of the progesterone induced binding factor (PIBF) by lymphocytes [43]. PIBF levels increase through pregnancy and drops post parturition. High PIBF levels promotes the differentiation of CD4^+^ T cells into Th2 [44, 45]. The Th2 cells secrete high levels of anti-inflammatory cytokines including IL-4, IL-5, and IL-10 which are crucial for maintaining a homeostatic environment in pregnancy [46, 47]. Aberrations in PIBF can result in altered immune-regulatory functions. The increase in pro-inflammatory cytokines such as IFN-γ and TNF-α can damage the placenta as well as the developing embryo [48].

While most craniofacial developmental anomalies (syndromic or non-syndromic) originate during the first trimester, they become diagnostically apparent only during the second trimester as tissues undergo maturation e.g., craniosynostosis. Endogenous estrogen is important for maintaining bone and cartilage. Mesenchymal stem cells exhibit estrogen receptors during chondrogenesis and facilitate their proliferation [49]. Exogenous estrogen and estrogen-like chemicals can disrupt normal craniofacial development [50]. Exogenous estrogen (Estradiol) has been shown to enhance the cytotoxicity of NK cells and increase the production of IFN-γ [51]. Similarly, estradiol has also been shown to enhance expression of pattern recognition receptors and toll like receptor-4 as well as stimulate low grade production proinflammatory cytokines such as TNF-α in peritoneal macrophages [52]. Zebrafish larvae exposed to exogenous estrogen showed craniofacial malformations [53]. Estradiol E2 has been shown to impair NCC migration [54], and affect several signaling pathways such as Bmp2a and Wnt [55].

During the second trimester, the corticotropin releasing hormone (CRH) also rises as the syncytiotrophoblasts increase in the placenta. At the same time, the hypothalamic pituitary axis (HPA) is upregulated. The rise in CRH levels results in an increase in the maternal cortisol levels [56]. Cortisol inhibits the progesterone's control of the prostaglandin-inactivating enzymes [57]. In a healthy pregnancy, a balance between these two hormones is important to control the inflammatory responses until the complete term. The rise in CRH through pregnancy is described as paradoxical; It exerts a protective anti-inflammatory effect on the HPA but also leads to an activation of the pro-inflammatory cytokines and keeps the maternal immune system primed by releasing debris from apoptosed extravillous trophoblasts into the maternal system [58].

The process of parturition, is an event marked by activated inflammation. Sacks (1998) compared the inflammatory changes during human parturition akin to sepsis [59]. The uterine membranes and the amniotic fluid become enriched with proinflammatory cytokines and prostaglandins, mediated by the innate immune system in preparation for labor [60, 61]. Signals of fetal maturity, such as fetal surfactant lung protein (SP) and stretch of the amniotic membranes, triggers parturition and birth [62]. It is hypothesized that preterm births due to infections and inflammation could be mediated via macrophages and the SP, however, the exact mechanism is yet unclear. Readers are referred to excellent reviews by Abu-Raya et al. [63] and Peterson et al. [64], which discuss the physiologically relevant inflammatory changes in each of the trimesters in greater details.

While several innate and adaptive responses tightly regulate the inflammatory states in pregnancy, such a state is also very delicate and sensitive to intrinsic and extrinsic environmental factors. Infections can affect all stages of pregnancy but with regards to craniofacial development, the first trimester is most critical. It is important to note that a physiologically regulated inflammation in pregnancy can prove a bane in presence of infections. In a non-pregnant state, minor infections are easily cleared by the body's response mechanism; In pregnancy however, due to the physiological “immunosuppression” the severity of responses to infections is increased and can become detrimental to both the mother and the developing fetus. In the following sections we will review such processes in greater details.

### Effect of Activated Maternal Immune Responses on Fetal Development

David Barker et al. hypothesized that low birth weight infants presented a higher risk of developing Schizophrenia and attention deficit hyperactivity disorder (ADHD) later in life [65]. This was one of earliest studies linking maternal inflammation to prenatal development as well as an exploration of developmental origins of psychiatric disorders. The hypothesis has evolved over the years and a role of the maternal inflammatory state in several other diseases has been recognized [66, 67]. Maternal immune regulation can be perturbed by several factors including nutritional deficiencies, infections, and risk factors such as smoking and substance abuse. However, the degree of effects observed, depends on whether the inflammatory trigger is acute or chronic. In this section, we will discuss both states, and focus mainly on the effect of dysregulated inflammation induced as a result of infections or dysbiosis in the maternal microbiome.

There are two main regulators of inflammatory responses in a pregnant state: hormones and immune cells. Acute inflammation as observed in infections, is typically short but marked by the infiltration of neutrophils, increase in monocytes, macrophages and pro-inflammatory cytokines [68]. Acute inflammatory reactions mediated by infections present with exaggerated responses and often, result in serious consequences [69]. Studies in pregnant Rhesus monkeys showed that transient chorio-decidual infection with Group B *Streptococci* induced a cytokine surge in the amniotic fluid resulting in a lung inflammation in the fetus at birth [70]. Cadarlet et al. showed that pregnant ewes injected with LPS, resulted in offspring with lung inflammation, reduced β-cell function, and impaired glucose metabolism [71]. While epigenetic changes will be discussed in greater details later, it is interesting to note that maternal infection has been shown to result in histone modification in fetal immune cells [72] thus priming them for future responses. Monocytes from purified cord blood cells of pre-term infants with exposure to chorioamnionitis were found to exhibit a hypo-responsive transcriptional phenotype to *Staphylococcus epidermidis* in a subset of genes involved in antigen presentation and activation [73].

Cellular receptors such as TLRs, Nod 1 and 2, and acute phase proteins such as C-reactive proteins (CRPs) are key to eliminating infections with the help of the complement system and the phagocytes. The binding of a pathogen activates the pathogen recognition receptor (PRR). PRRs activate an inflammatory cascade resulting in the release of cytokines, matrix metalloproteinases and other growth factors. Proinflammatory cytokines such as interleukin-1β, 6 and 8 as well as TNF-α stimulate both the release of matrix metalloproteinases as well as prostaglandins, activating the damaging inflammatory response [74, 75].

A balance in the levels of estrogen and progesterone is very important to the maintenance of pregnancy. Progesterone plays a key role in the inhibition of proinflammatory cytokines and prostaglandins. Unfortunately, this “immunosuppressive” effect is disadvantageous to the mother as the severity of response to infections is often exaggerated. While the exact mechanism is unclear it is hypothesized that this effect might be due to two reasons: one, increase in the levels of progesterone increases susceptibility to infections in pregnancy [76]; and second, while inflammatory responses are necessary to clear pathogens, due to the immunological landscape of pregnancy, the severity of infections are increased. Pregnant mice infected with influenza were found to have greater viral replication in their lungs compared to the non-pregnant females with higher levels of TNF-α, CCL2, and 3 as well as CXCL1 [77]. Pregnant mice infected with *Toxoplasma gondii* showed improved outcomes for those treated with recombinant IFN-γ [78].

Chatterjee et al. used inbred guinea pigs infected with guinea pig CMV (GPCMV), to study the effect of immunization with anti–glycoprotein B (gB) antibodies during pregnancy. The study showed that immunization with hyperimmune anti-gB antibody in early pregnancy reduced both the incidence and the severity of newborn GPCMV infection and prevented growth retardation [79].

Chronic inflammation in pregnancy is often a result of long-persisting, low-grade infections or long-term reaction to an inflammatory stimulus. In pregnancy, chronic inflammation is most observed with systemic diseases in the mother such as obesity, diabetes, hypertension, chronic stress, or low-grade viral infections. Such an inflammatory reaction is marked by the recruitment of monocytes and lymphocytes with tissue damage and may have long-term effects in the developing fetus. Studies have shown that chronic inflammation in the mother is associated with long-term neurodevelopmental disorders in the offsprings [80, 81].

**Figure 2:**
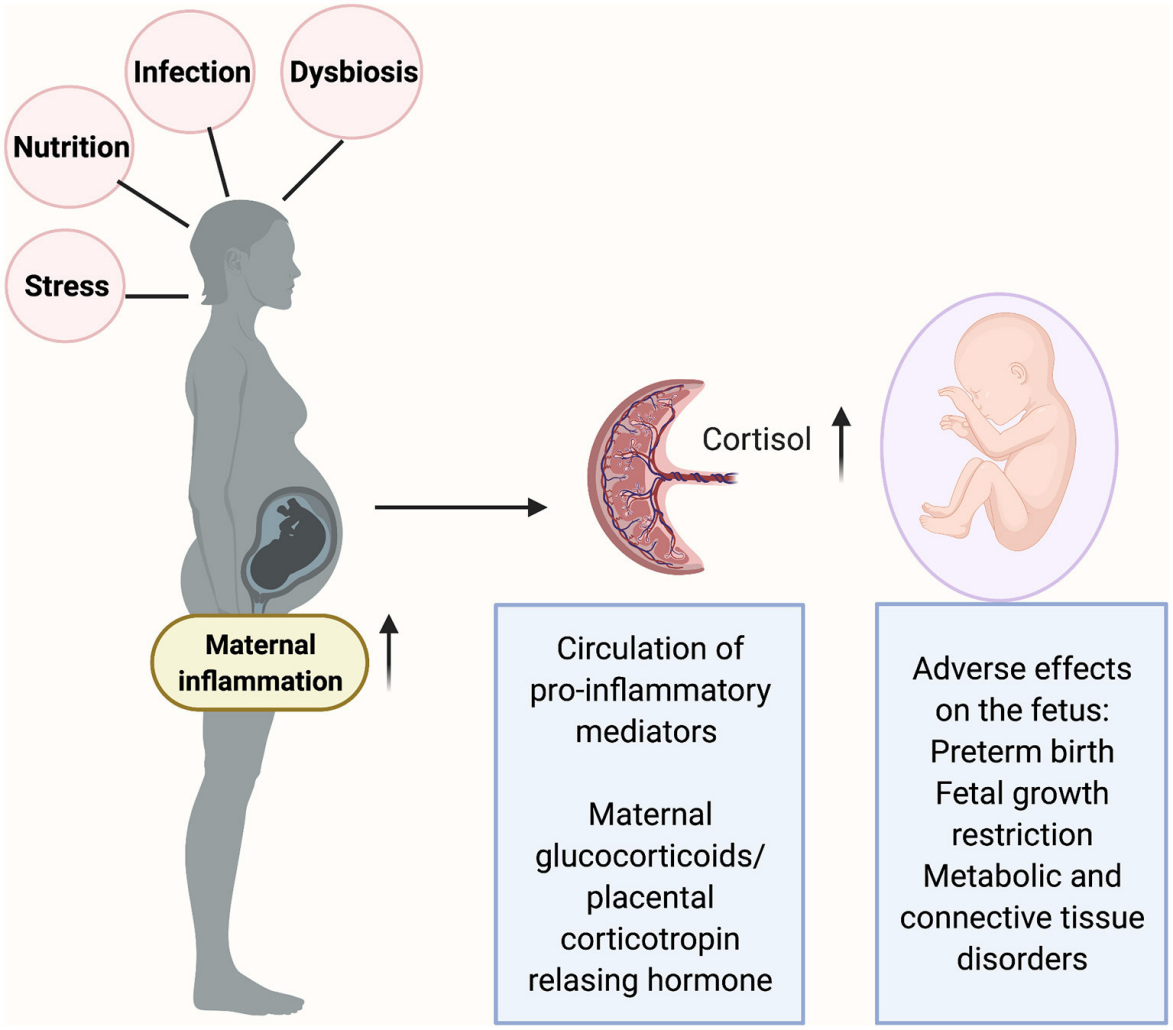
Inflammatory triggers in pregnancy such as maternal infection, stress and malnutrition can cause a release of pro-inflammatory mediators at the maternal-fetal interface (placenta). The placenta produces corticotropin releasing hormone (CRH) in response to stress. Excessive inflammation can result in fetal exposure to the glucocorticoids which in turn can reprogram the fetal hypothalamic-pituitary-adrenal (HPA) axis and alter the fetal developmental processes as well the immune system development. Figure created using the BioRender software.

Though a direct role has not been established, it may be hypothesized based on the current scientific evidence that, maternal inflammation (induced by infections) can prime the fetal immune system as well as adversely affect the hypothalamic-pituitary-stress axis (HPA) resulting in long-term effects on fetal development (Figure 2). In Rhesus monkeys, blood cells from juvenile offspring of stress induced mothers exhibited reduced levels of TNF-α and IL-6 when treated with LPS *in vitro* [82]. Rats exposed to prenatal stress exhibited an altered thymic function coupled with a decrease in total lymphocytes as well as in ${\text{CD}}_{4}^{+}$ and ${\text{CD}}_{8}^{+}$ lymphocytes [83, 84]. Glucocorticoids are released when the mother is exposed to stressors. Glucocorticoids are lipophilic and thus theoretically, can cross the placenta. While the levels of glucocorticoids reaching the fetus may be small, it may have significant effects on overall development of the fetus. Fetuses and preterm newborns exposed to chronic intrauterine infections have been shown to exhibit elevated amniotic fluid cortisol levels [85] and increased cord blood cortisol levels [86]. Such an increase can persist through the first 3 weeks of neonatal life [87].

Exposure of the fetus to glucocorticoids has also been shown to impair muscle growth and skeletal muscle mass [88]. Skeletal growth and development are significantly affected by both infection and chronic inflammation [89, 90]. Pregnant Sprague-Dawley rats injected with bacterial endotoxins resulted in impaired fetal myoblast function, increased protein catabolism, and reduced skeletal muscle growth near term [71]. Embryoid bodies exposed to bacterial LPS resulted in the inhibition of germ layer differentiation [91]. Similarly, Rubella virus infected human induced pluripotent stem cells demonstrated several epigenetic modifications as well as impaired germ layer differentiation [92]. Maternal stress during gestation resulted in fetal growth restriction likely through increased cortisol levels. This was found to be linked to altered muscle growth and skeletal development in both human and animal models [93, 94]. Exposure to corticosteroids during pregnancy has been associated with the risk of developing orofacial defects in animal models however, their effects in humans remain unclear [95, 96].

Along with immunological factors, several other materno-fetal communication “factors” have been identified. Circulating material derived from the fetoplacental unit such as the extracellular vesicles (EVs) [97, 98], microparticles [99], cell-free fetal DNA (cffDNA) [100] as well as fetal microchimeric cells [101] have been shown to play an important role in both health and maternal inflammation state. Extracellular vesicles have been shown to mediate communication between cells by serving as vehicles for proteins, lipids, and microRNAs (miRNAs) and are postulated to play a role in embryo implantation [102], placentation [103], and maintenance of pregnancy [104]. ECVs have also been implicated in the pathogenesis of preecclampsia [105] and preterm births [106], and are touted as prognostic biomarkers [107]. While important, the role of ECVs in pregnancy is yet not fully understood [103, 108, 109].

Microparticles are detected in context of oxidative stress and are hypothesized to be mildly pro-inflammatory in function, though their function remains yet unclear [110]. cffDNAs are derived from apoptotic trophoblasts and their concentration increases with pregnancy. They have been shown to be hypomethylated, thus making them amenable to agonism by TLR 9 [111]. It is hypothesized that cffDNA may serve to prime the TLR-dependent maternal immune responses. However, further studies are needed to establish this. Fetal microchimeric cells (cells transmitted from fetus to mother) have been shown to persist in the maternal system long after gestation [112]. They have been identified at tumor sites including breast cancer [113], thyroid cancers [114] as well as melanomas [115]. While the role of fetal cells is not fully understood, these findings show that while the maternal immune system programs the fetal systems, a reverse mechanism is also simultaneously at play i.e., the fetal molecules also reprogram the maternal immune system.

Studies on maternal inflammation have provided significant insights on the etiology of developmental defects in the fetus but are limited in scope due to significant technical and ethical concerns. Most of the available data has been obtained in animal models with the introduction of systemic infections or sepsis, which are rare occurrences in human pregnancies. Other studies have examined the cause and effect using medical records which includes the risk of exclusion of contributing factors such as diet, gut microbiota, and metabolism. Further research is needed in large population groups of mothers experiencing activated inflammation in pregnancy and adverse developmental outcomes in offspring to examine the association more robustly.

### Pregnancy and Infections

Developmental pathways are complex and depend on both internal and the external signals. Microbial colonization begins in the early stages of life and plays a role in several of these developmental pathways [116]. Most evidence for a direct role of infection in pregnancy comes from bacteria that have been shown to traverse the intact materno-fetal membranes and are hypothesized to have originated from the lower genital tract [117]. However, a study on the ecological succession of the vaginal microbiota through the course of gestation by Rasmussen et al., observed that the vaginal microbiota represents only a small portion of the microbiota in the newborn [118]. Thus, suggesting that the neonate must receive microbes from other sources [118]. Pathogens and their molecules have now been shown to traverse the placenta. Despite these, the sterility of the environment surrounding the fetus has been highly debated [119]. Studies have shown that pregnant women exhibit microbiome profiles distinct from the non-pregnant control groups [120–124]. In this section, we will discuss the existing evidence regarding the possible role of microorganisms from three key sources: the placenta, the vagina, and the oral cavity.

#### The Placenta

In 1964, Billingham proposed a bi-directional transfer of cellular elements from the placenta which was a paradigm shift for understanding maternal-fetal interactions [1]. While the placenta indeed allows for a bi-directional transfer of molecules, it also serves as a robust barrier against fetal infection by most pathogens [125]. A study by Aagard et al. reported that a normal placenta may harbor its own microbiome [126]. This study also reported the similarity of the isolated phyla to those found on other locations in the body such as the oral cavity, vagina, and the gut [126]. However, the studies that followed, argued both for and against this finding [127–130]. Largely, in the absence of inflammation, the placental microbiome has been described as having a low-abundance and low-biomass. The argument against a placental microbiome centers around possible contamination, systemic infections, and the detection of dead bacteria as whole genome sequencing does not differentiate live from dead [131]. The most recent and compelling evidence in favor of the placental microbiome was a study by Younge et al. [132], where the authors used a mouse model to demonstrate cultivable bacteria from the fetal gut. These bacteria were only isolated in the second trimester and could not be isolated in the third, suggesting that the bacteria could have somehow traversed the placenta to colonize the fetal gut. Further, several studies have reported the association of placental dysbiosis with adverse pregnancy outcomes such as preterm births or premature rupture of membranes [128, 133]. Villitis, an inflammation of the chorionic villi of the placenta caused by infections, can result in miscarriages [134] or adverse fetal outcomes [135, 136]. While the exact mechanism for placenta mediated adverse effects is unclear, several theories have been proposed. One proposed mechanism is that trophoblasts in the first trimester express TLRs (such as TLR-2), which can bind to bacterial endotoxins and peptidoglycans. This can shift the Th-1 balance to a pro-inflammatory state [137]. In dizygotic twins with dichorionic placentas and chronic villitis, the twins exhibited growth differences corelating to the severity of villitis for each placenta [138]. The placenta with higher villitis also showed extensive T-cell infiltration as compared to the non-affected placenta [138].

Viral infection in pregnant women is associated with adverse pregnancy outcomes, including stillbirths and congenital anomalies in the fetus [139–141]. Interestingly, the viral entry mediators such as heparan sulfate, herpesvirus entry mediator A (HveA), HveB, and HveC are not expressed on the syncytiotrophoblasts but only on the extravillous trophoblasts [125, 142]. Despite this, CMV and HSV have been detected in the maternal decidua in both symptomatic and asymptomatic mothers [143–146]. Using mouse models of viral infections, it was shown that the Zika virus in pregnant mice resulted in damage to the placenta, microcephaly, and fetal demise. The authors also showed that the Zika virus exhibited placental tropism [147]. Recently, studies on SARS-CoV-2 infection in pregnant women has shown that the virus causes placental infection, inflammation, and eventually, neurological complications in newborns [148]. This study was one of the many other studies supporting the role of SARS-COV-2 infection in mediating maternal inflammation in pregnancy [149, 150]. While the data on the presence and effect of SARS-CoV-2 in vaginal secretions, amniotic fluid, and breastmilk in infected women is still accruing, studies have reported a concerning role for the virus in pregnancy [151–155]. Further studies are needed to confirm vertical transmission as well as examine its long-term effects in neonates. Thus, while the exact mechanism is yet unclear, it is likely that the viral-mediated maternal immune activation and epigenetic modifications may cause the adverse effects observed in the embryo.

#### The Vagina

While there are geographical and inter-individual variations in the vaginal microbiota of pregnant women, the consensus remains that, healthy pregnancies are associated with a greater load of *Lactobacilli* [121, 156, 157]. *Lactobacilli* were described as colonizers of the vaginal microbiota in 1892 by Albert Döderlein and were hypothesized to play a role in maintaining pH and preventing colonization of other pathologic species [158]. A study in pregnant women in rural Malawi showed that a *Lactobacillus* deficient vaginal microbiota was associated with shorter term pregnancies. A subset of the cohort in the same study also exhibited an abundance of *Peptostreptococcus anaerobius* and associated shorter term pregnancies and newborns with lower length for age Z-score [159]. A study using pregnant rats showed that the weights of placenta and the offspring from rats infected with *Escherichia coli* (observed as a co-colonizer with *Lactobacilli*) were significantly lower as compared to the non-infected controls [160]. Bacterial dysbiosis in the lower genital tract of pregnant females commonly results in bacterial vaginosis. The pathogens causing vaginosis may vary depending on the cause from *Gardnerella vaginalis, Bacteroides, Peptostreptococcus, and Prevotella* [161]. Bacterial vaginosis is associated with an increased concentration of endotoxins in cervical mucus or the vaginal fluids [162]. Endotoxins are components of the Gram-negative bacterial cell wall. A study by Kamiyama et al. [163] reported that among women undergoing *in vitro* fertilization (IVF), a successful pregnancy did not occur if the levels of endotoxin concentration in the menstrual fluids was >200 pg/ml [163]. Bacterial vaginosis has also been shown to result in preterm births, low birth weight of newborn and/or restricted growth of the fetus and sometimes, miscarriages [164–167].

Sexually transmitted diseases (STDs) can result in complications during pregnancy. Maternal gonorrhea is associated with low preterm birth weight, premature rupture of membranes and chorioamnionitis [168–170]. Pregnant rats infected intraperitoneally with *Neisseria gonorrhoeae* showed a materno-fetal transmission resulting in fetal mortality [171]. Furthermore, studies investigating birth defects and maternal genitourinary infections through the first trimester observed that STDs in pregnancy were associated with significant developmental defects in the newborns [172, 173]. These studies support the theory of vertical transmission via the maternal genitourinary tract for eliciting adverse effects on the fetus.

Studies on the vaginal mycobiome have identified *Candida* and *Saccharomyces* as the predominant genera in pregnancy [174]. Fungal infections can result in chorioamnionitis, an inflammation of fetal membranes, an important risk factor for low birth weight, preterm births, and neurodevelopmental defects in the newborn [175–177]. The vaginal virome has been poorly identified due to the difficulties in isolation owing to small viral genomic material and ongoing mutations. However, *Herpesviridae, Papillomaviridae, Polomaviridae*, and *Parvoviridae* have been isolated routinely [30]. Though studies of the vaginal virome have not been able to identify specific viruses associated with adverse pregnancy outcomes such as preterm birth, quantitatively, the vaginal viral loads are higher in those who experienced such adverse outcomes [178–180].

#### The Oral Cavity

A healthy pregnant oral microbiota has been identified to primarily belong to *Actinobacteria, Bacteroidetes, Chlamydiae, Chloroflexi, Firmicutes, Fusobacteria, Gracilibacteria (GN02*), *Proteobacteria, Spirochaetes*, SR1, *Synergistetes* and *Saccharibacteria* (TM7) [181]. Systemic changes in the human body has been shown to exert significant influence on the diversity and the richness of the oral microbiota [182]. Studies have reported a difference in oral microbiome between pregnant and non-pregnant states. Balan et al. reported a difference in bacterial abundance in pregnant patients between the second and the third trimester. There were also significant differences in taxa between the individual patients [183]. *Prevotella negrescens* was observed to increase in abundance in periodontal plaque samples during the second trimester [184]. Recently, a study in Japanese women observed an increased abundance of *Porphyromonas gingivalis* and *Aggregatibacter actinomycetemcomitans* during early and mid-pregnancy, compared to non-pregnant groups [185]. *Candida* species were found to be more abundant during mid and late pregnancy [185]. In contrast, DiGiulio et al. observed no significant difference between change in bacterial abundance across and post-pregnancy [157].

Periodontitis is the inflammation of tissues surrounding the teeth mediated by a dysbiosis of dental microbial biofilms. Pregnant women are predisposed to developing periodontitis by the virtue of hormonal changes. It is suggested that women with periodontitis during pregnancy may be at a heightened risk for adverse pregnancy outcomes though the exact mechanism is unknown [186]. Previous studies have identified *P. gingivalis* and *Fusobacterium nucleatum* in the amniotic fluid of pregnant females at risk for premature delivery [187, 188] as well as in the placentas of patients with preeclampsia [189, 190]. An abundance of *Campylobacter rectus, F. nucleatum*, and *P. gingivalis*, have been associated with adverse outcomes for pregnancy specially if associated with contributing systemic diseases such as diabetes or hypertension [191]. These periodontal pathogens are suggested to mediate their systemic effects by hematogenous dissemination. Increased levels of estrogen and progesterone enhances the permeability of the dental junctional epithelium facilitating the dissemination of periodontal pathogens [192]. *P. gingivalis* and *T. denticola* have also been shown to internalize in several types of host cells [193, 194] resulting in altered immune responses as well as mediating epigenetic changes.

*Bergeyella*, a genus of oral bacteria, isolated from amniotic fluid and the vaginal flora of pregnant women was genetically similar to the clones isolated from the mother's oral subgingival flora, suggesting multiple sources of origin [195]. *F. nucleatum* has been detected in a wide variety of placental and fetal compartments including amniotic fluids, fetal membranes, cord blood, and neonatal gastric aspirates [179, 196–198].

Syphilis is caused by *Treponema palladium* and its effect in pregnancy has been investigated from the early 1900's. The route of transmission for *T. palladium* is highly debated. Spirochetes are known to colonize both from the lower genitourinary tract and the oral cavity. They have also been shown to cross the placentas as early as 9–10 weeks [199–201]. Maternal syphilis has been known to result in preterm births, non-immune hydrops fetalis, spontaneous abortion, and stillbirths [157]. Several consequences for the surviving fetus include growth restriction, orofacial defects, anemia, thrombocytopenia, hepatomegaly, and hydrops fetalis [157, 202–207].

While a direct mechanism for pathogen transmission and adverse outcomes in pregnancy yet remain to be established, studies have definitely highlighted an association. Further, studies are also lacking in several areas of the pregnancy microbiome such as the virome, fungal diversity as well as their metabolites. While reported independently, a cross-species talk and commensalism between different pathogens cannot be ignored. Often such mutualistic associations between pathogens in a dynamic landscape such as pregnancy shapes disease outcomes and thus, must be examined in-depth. *Bifidobacteria* are amongst the first colonizers of neonatal gut microbiome and play a key role in shaping their immunity [208, 209]. *Bifidobacteria* strains isolated from maternal breast milk, vaginal, and fecal samples of mother and child combinations have been found to be identical, indicative of multiple transmission routes from the mother to infants [210–212]. *Bifidobacteria* also serves as carrier of (pro)phages which is important for establishing neonatal virome. The SARS-COV-2 virus has been shown to be associated with enhanced bacterial co-infections [213]. Additionally, SARS-COV-2 binds to the highly expressed oral Angiotensin-Converting Enzyme-2 (ACE-2) receptors [214]. ACE-2 is also highly expressed in the placenta, uterus, and the materno-fetal interface [215, 216]. Fetal ACE-2 plays a key role in the development of the heart, lungs, and brain [217]. The role that this interaction plays in pregnancy and fetal development is yet to be determined.

**Figure 3:**
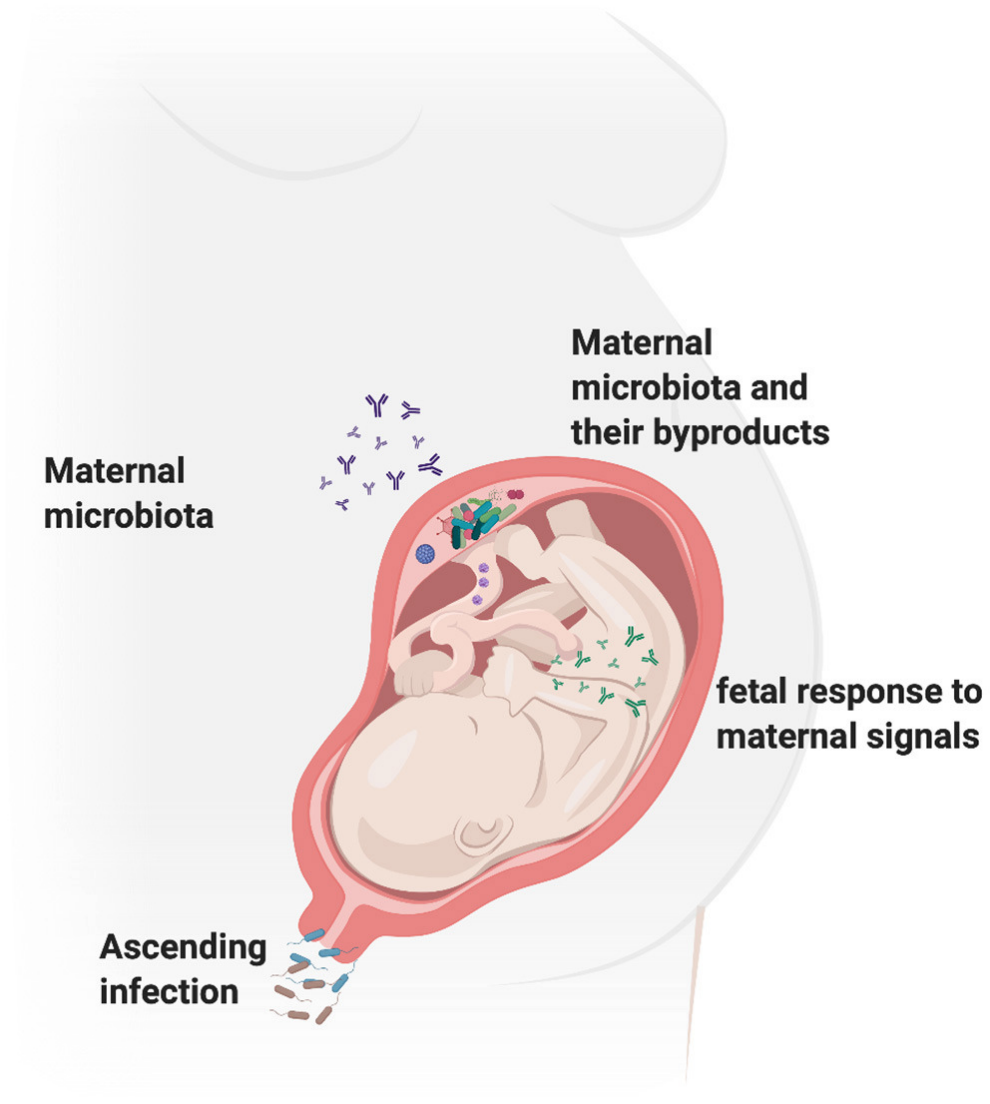
Gestational environment shapes fetal programming. The pregnant vaginal microbiome is known to consist of more than 170 species and studies suggest that the vagina might be a source of microbes for the fetus via vertical transmission *in utero* or parturition. Maternal microbiome at sites such as the gut and the oral cavity have also been shown to contribute to the development of normal fetal microbiome and mucosal immunity. Bacteria, their metabolites and other by-products have been isolated in the placenta and are also shown to shape fetal immune development post-birth. Thus, the maternal microbiome can impact the fetal development via several mechanisms including altering the maternal inflammation, as well as mediating direct effects on fetal genetics. Figure created using the BioRender software.

Figure 3 outlines the most commonly known routes of fetal infection based on the discussed evidence. Overall, recent studies collectively lean toward a role for microbes in fetal development. However, the evidence for the adverse effect of microbes on fetal development point more toward a role of inflammation in the same and further studies are needed to understand and clarify this argument.

## Pathogens as Epigenetic Modifiers

Epigenetics is the study of changes or modification in a gene function that are transmitted to the daughter cells without altering the sequence of the respective gene. The epigenetic modification of a specific chromosome in the gametes or zygotes results in the differential gene expression in the somatic cells also known as genomic imprinting [218, 219]. Epigenetic mechanisms are complex and include processes such as methylation, acetylation, phosphorylation, ubiquitylation, and sumoylation of DNA or the post-translational modification of histones. Of these processes, methylation of DNA is the most well-studied mechanism in which a cytosine base in position of C5 in CpG dinucleotides (followed by guanine), undergoes methylation (addition of CH_3_) [220, 221]. Methylation of DNA is heritable, and relatively stable as compared to histone modifications. Recent studies suggest that pathogen mediated DNA methylation occurs fast and regulates the expression of host genes involved in immune responses [222, 223]. This is carried out by a group of enzymes known as DNA methyltransferases that transfer a methyl group from S-adenyl methionine (SAM) to the fifth carbon in the cytosine base to modify as 5-methyl cytosine. This results in chromatin condensations and disrupted interactions between DNA and the transcription factors. DNA methyltransferases (*DNMT1, DNMT3A*, and *DNMT3B*) are expressed and involved in the development of the embryo. The expression of these enzymes is reduced in terminally differentiated cells except for the post-mitotic neurons in the human brain [224, 225]. The diseases caused by fetal epigenetic reprogramming are uniquely regulated and the resulting phenotypes are often delayed (i.e., the effect during fetal exposure is long lasting). While the reprograming of fetal genetic material begins during prenatal development, it continues through the lifetime of an individual [226]. Though not fully understood and limited, there is growing evidence to suggest that microorganisms and their byproducts can mediate epigenetic changes during prenatal development which can contribute to future diseases [227, 228].

While the amniotic fluid is generally believed to be sterile, studies have reported that at least 1% of the women present with subclinical microbial invasion of the amniotic cavity (MIAC) [229]. The majority of sub-clinical MIAC is endogenous in origin. Few of the isolated species are composed of Mycoplasmas (*Ureaplasma spp*.) and oral bacteria such as *Fusobacterium* and *Streptococcus* [230]. While a direct role of these microorganisms on the fetus remains to be explored, studies have shown that they are capable of mediating epigenetic changes.

Internalization is an important mechanism used by several pathogens for dissemination and immune evasion. Internalization of oral pathogens such as *P. gingivalis* and *T. denticola* in dental follicle stem cells has shown to reduce the secretion of IL-10 and decreased chemotaxis of PMNs [231]. Though not shown in pregnancy, studies have reported that *P. gingivalis* [232] as well as their lipopolysaccharides, can downregulate the expression of DNA methylases *DNMT3A* and *DNMT1* in epithelial cells [233, 234]. Infection of periodontal ligament cells with *T. denticola* increased MMP-2 expression and altered genes encoding for chromatin modification [235]. DNA methylation in periodontitis is not restricted to the site of infection. Increased methylation of the promoter regions of TNF [236] and IL-6 [237] genes have been reported in blood cells, in patients with periodontitis. Further, *F. nucleatum*, was found to translocate to the placenta in pregnant mouse models and cause adverse outcomes in pregnancy [238]. Independent studies have also associated *F. nucleatum* with DNA methylation in colorectal cancer and modulate autophagy [239–241] highlighting its role in modulating epigenetics.

*C. rectus*, another key periodontal pathogen was found to transverse the fetoplacental unit in pregnant mouse models and cause growth restriction in the fetus [242].

Using mice infected with *C. rectus*, Bobetsis et al. reported DNA hyper-methylations in several regulatory genes in the fetus [243]. This study identified a significant downregulation of insulin-like growth factor 2 (IGF2) as a result of increased methylation of the IGF promoter upon placental infection with *C. rectus*. Eleven of the six CpG methylation sites in IGF2 exhibited hypermethylation upon infection with *C. rectus* leading to intra-uterine growth restricted placentas. Studies using fetal cord blood cells in pre-term infants revealed significantly increased levels of methylation of pleomorphic adenoma gene 1 (PLAG1) as well as the Paternally expressed gene 3 (PEG3) [244, 245]. PLAG1 encodes a developmentally regulated, SUMOylated and phosphorylated zinc-finger transcription factor while the PEG3 genes are important in muscle and neuronal lineage development. In African populations, maternal HIV infection was found to be associated with increased methylation of PEG3 gene in the neonates [246]. Metabolites by pathogens have also been shown to mediate epigenetic effects. Folate produced by *Bifidobacterium* and *Lactobacilli* generate SAM, resulting in DNA methylations in the host cells [247]. Short chain fatty acids (SCFAs) are a part of free fatty acids generated by bacteria as their metabolic by-products. Patients with severe periodontitis exhibit high SCFA levels in dental biofilms. *P. gingivalis* derived SCFAs have been shown to induce reactivation of the Epstein-Barr virus and Kaposis's sarcoma associated herpesvirus [248]. This was associated with an increased host histone acetylations and transactivation of viral chromatin [248]. Similarly, Aflatoxins, produced by fungal species commonly found on foods, are also known to be associated with inducing DNA methylations at 71 CpG sites in infant white blood cells [249] and growth faltering in the 1^st^ year [250].

Chorioamnionitis (CA) is marked by an infiltration of the amniotic sac by the maternal neutrophils as a result of infection, leading to a fetal inflammatory response syndrome [251]. DNA methylation patterns in chorionic villi, amnion, and chorion of CA and non-CA patients [244, 252] showed DNA methylation sites close to the promoter regions of immune-response genes such as *HLA-E, CXCL4, RAB27A, IRX2*, and *HSD11B2*. In neonatal monocytes, the promoters of innate-immunity genes *IL1B, IL6, IL12B, TNF*, and *CCR2* exhibit significantly higher histone-methylation modifications [253, 254]. These monocytes under CA conditions express significantly lower levels of pro-inflammatory cytokines IL-1β, IL-6, IL-8 that might predispose the fetus to sepsis upon secondary infections [72].

The infection of bladder epithelial cells with *E. coli* in pregnant women can cause hypermethylation of tumor suppressor gene *cyclin dependent kinase 2A* (CDK2NA) and result in epithelial dysplasia [245, 255]. CDK2NA is also methylated in HPV-16 induced epithelial dysplasia. Though exceedingly rare but a significant risk factor in pregnancy, the food born pathogen *Listeria monocytogenes* is known to cause histone modifications. Such modifications can lead to transcriptional activation of MAPK pathways and affect histone modifications in chemokine genes (C-x-C motif) *Cxcl2* [256, 257] predisposing the fetus to future immune associated diseases.

It is interesting to note that pathogen mediated DNA methylation is also an effective mechanism for evading immune responses. Addition of methyl donor S-adenosylmethionine (SAM) reduced LPS-mediated inflammatory response in RAW 264.7 macrophages [258]. Similarly, bovine dermal fibroblasts treated with demethylating, and hyper-acetylating agents demonstrated altered responses to LPS stimulation [259]. These studies support the possibility that subclinical chronic infection with pathogens can mediate subtle epigenetic changes, though studies are needed to confirm such an effect in pregnancy.

Gestation is a period of genetic reprogramming in the life of an individual and understanding the role of healthy maternal microbiome vs. a dysbiotic one in fetal development is important. Infection with different types of pathogens mediates different epigenetic effects both transient and stable. Current understanding of the role of microbes in epigenetic modification is limited but ever evolving. Given the differences in human and animal models of infections, further studies are needed to understand infection and inflammation by specific pathogens in the context of epigenetics and pregnancy outcomes. Table 1 lists some key epigenetic modifications by maternal infections and their associated fetal outcomes.

**Table 1:** Epigenetic modifications on human genes induced by maternal infections and associated fetal adverse outcomes.

**Gene**	**Epigenetic modification**	**Associated micro-organism/maternal disease**	**Fetal outcome**	**References**
*IGF2*	Hypermethylation of promoter region 0	*C. rectus*	Restricted intra-uterine growth	[243]
*PLAGL1*	DNA methylation	Chorioamnionitis	Pre-term birth, pheochromocytoma, capillary hemangioma, transient neonatal diabetes mellitus	[244, 260–262]
*IL1B, IL6, IL12B, TNF, and CCR2*	Histone modification (H3K4me3)	Chorioamnionitis	Alteration of innate immune pathways	[253]
*CDK2NA*	Hypermethylation	*E. coli*	Epithelial dysplasia	[245, 255]
*MAPK*	Histone modification	*L. monocytogenes*	Immune dysfunction	[256, 257]
*CXCL2*	Histone Modification	*L. monocytogenes*	Immune dysfunction	[257]

## Maternal Infections, Fetal Neurogenesis and Craniofacial Defects

Craniofacial development involves complex series of events mediated by cells derived from all three germ layers and the NCCs. The cranial structures include the brain, eyes, ears, nasal, and gustatory apparatuses. The facial structures involve the development of the jaws, teeth, and their associated structures. While genetics is the primary determinant, multivariant signaling and temporal organization are required for the normal development of these structures. Any subtle perturbation in the events above can cause defects in the development of craniofacial structures.

Craniofacial birth defects are the commonest birth defects next only to congenital cardiac defects.

Craniofacial anomalies are typically termed to include cleft lip and/or palate, defects of the central nervous system, eye, jaw, and dental defects. In this section we aim to briefly discuss the studies associating maternal infections and structural development of key craniofacial defects such as neural tube defects, cleft lip/palate, and dental anomalies. Table 2 lists key organisms and the studies associating them with congenital craniofacial defects in newborns.

**Table 2:** Key organisms associated with congenital craniofacial defects in the newborn.

**Causative agent**	**Organism**	**Defect**	**References**
Virus	Influenza, Rubella, Cytomegalovirus, Epstein-Barr, Coxsackie and hepatitis B viruses	Orofacial clefts (Cleft lip with or without cleft palate)	[263–265]
	Zika virus	Brain abnormalities (with or without microcephaly) Eye abnormalities Cleft palate Micrognathia Hypodontia Delayed dental eruption	[266, 267]
Bacteria	*Treponema pallidium* (Syphilis)	Cleft lip Hutchinson's triad Moons/Mulberry molars	[207, 268]
	*Chlamydia Neisseria gonorrhea* (pelvic inflammatory disease)	Cleft lip with or without cleft palate.	[172, 173, 269]
Fungi	*Candida albicans C. glabrata* (Vulvovaginal candidiasis)	Chorioretinitis/Cerebral candidiasis	[270, 271]
Parasite	*Toxoplasma gondii*	Chorioretinitis	[272]
	*Plasmodium spp*.	Preterm birth Intrauterine growth restriction Craniofacial anomalies	[273]

### Neural Tube Defects (NTDs) and the Development of the Brain

The research on maternal inflammatory states and infection on the development of neural tube structures and brain development are still ongoing. A strong correlation between the maternal nutritional status, specifically Iron, Magnesium, vitamin B6 and B12 deficiency and the development of neural tube defects in newborns has been reported [274, 275]. *Helicobacter pylori*, an oral and gastrointestinal pathogen [276], was recently identified in the maternal vaginal microbiome with yeast as a carrier [277]. Maternal *H. pylori* infection is correlated to the risk of developing pre-eclampsia, spontaneous preterm birth, and intrauterine growth restriction (IUGR) [278]. *H. pylori* infection is associated with decreased folate, vitamin B12, and ferritin bioavailability to the fetus, and thus, may play a role in the development of NTDs [274, 279]. Maternal syphilis is associated with the development of hydrocephalus in the newborns [280] along with other defects. Viral infections such as maternal varicella-zoster virus infections have been shown to be associated with fetal hydrocephalus, porencephaly, hydranencephaly, calcifications, polymicrogyria, and focal lissencephaly secondary to necrotizing encephalitis [281, 282]. Similarly, maternal hepatitis B and C are also associated with adverse neurological development in the fetus, although more mechanistic studies are needed [283].

### Cleft Lip and Palate

Orofacial clefts (OFC) are one of the most widely known and common craniofacial anomalies in newborns [284]. These are characterized by the failure of fusion of facial processes seen overtly as a space or a gap in the upper lip, alveolus, or palate. OFC can be divided into three categories: cleft lip (CL) only, cleft palate (CP) only, and cleft lip associated with cleft palate (CLP). Further, clefting can occur either as isolated (non-syndromic) or as a part of a syndrome with other symptoms. OFC is caused by a combination of environmental factors (e.g., maternal illness, smoking/alcohol consumption, and malnutrition), and genetic predisposition [285, 286]. Studies have reported an association between maternal hyperpyrexia, illnesses, and infections with the occurrence of CLP in the newborns with no gender differences [287], though the mechanism is poorly understood.

Norman Gregg reported a causal association between maternal rubella infection and the occurrence of defects such as OFC in newborns in 1948 [288]. It is now hypothesized that maternal rubella results in an altered hepatic metabolism of vitamin A resulting in the manifestations of the congenital rubella syndrome [289]. A study in Latin-America showed that maternal exposure to acute (influenza) and chronic (syphilis) infections were significantly associated with the incidences of cleft lip cases [263]. The authors hypothesized that the observed association between influenza and cleft lips could be due to the vascular disruption during embryonic period caused by hyperthermia and/or the use of salicylates and may not be directly an effect of the viral infection [263]. Maternal exposure to genitourinary infections has also been associated with cleft lip in the offspring. This association was stronger if the mother reported an exposure to *Chlamydia* [173]. While the mechanisms involved in the association between chlamydial infection and OFC are not well-characterized, it has been suggested that the chlamydial 60 kD heat shock protein (hsp60), a potent inducer of inflammation, could affect pregnancy outcomes because serum anti-hsp60 antibodies may interfere with the development of the embryo [290]. Maternal toxoplasmosis has also been reported as a causative factor for the development of anophthalmia with oro-orbital and parasagittal clefts in the newborn [272].

Several genes have been identified to be associated with OFCs, however, in terms of gene-environment interaction, the most well-studied genes are the interferon regulatory factor-6 (*IRF6*) and the poliovirus receptor related-1 (*PVRL1*). These two gene families are known to modulate immune responses to infections. IRF6 belongs to the IRF gene family which are known to regulate expression of interferons after a viral infection. IRF6 has been shown to be expressed in the maternal endometrial cells as well as the trophectoderm of the fetus [291]. Mutations in the IRF6 gene have been shown to result in Van der Woude's syndrome characterized by cleft lip/palate [292]. The *PVRL1* gene also known as the *Nectin 1* [293] gene is known to serve as one of the three primary receptors for the alpha herpes virus binding and entry or the herpes virus entry mediator C (HvecC) [294]. Mutations in the *PVRL1* gene have been associated with sporadic non-syndromic cleft lip/palate in newborns [295, 296]. A recent study has shown the association of DNA methylation for palate forming genes and non-syndromic cleft lip/palate in both a Brazilian cohort as well as a UK cohort suggesting epigenetic modifiers as contributors to OFC [297]. While an abundance of mechanistic insights exists in term of the role these genes play in palate development, further studies are needed to understand the effect of maternal infections as epigenetic modifiers for these genes.

### Dental Anomalies

Dental development begins around 6 weeks post-fertilization with the migration of neural crest cells. As described before, NCCs are highly sensitive to environmental changes and their migration and differentiation is mediated mainly by signals from the surrounding tissue [27, 298]. The dentin and functional ameloblasts for the deciduous and permanent teeth begin activity around 18 and 32 weeks, respectively. Dental anomalies can occur as structural (morphogenetic) defects or a complete absence of a few (oligodontia, hypodontia) or all teeth (anodontia). The occurrence of dental defects has been linked to several causative agents including maternal stress, smoking, alcohol use, hyperpyrexia, and use of antibiotics or other teratogenic causes [299, 300]. Occurrence of dental defects, similar to OFCs, can exist both in isolated forms and as a part of a syndrome. Several studies have reported a correlation between preterm births and lower birth-weight to be associated with delayed tooth eruption [301]. Further, studies have also found that very low birth weight infants are at a higher risk for dental caries and developmental defects of teeth as compared to full-term (term) babies [302–304]. No direct associations between pathogens, epigenetic effects and dental defects have been fully established so far. Thus, we have included a discussion of reports regarding maternal infections and associated dental defects.

Maternal rubella can result in tooth agenesis as well as other morphologic dental abnormalities [305]. Congenital syphilis results in a syndromic dental morphogenic abnormality known as Hutchinson's teeth [268] which is characterized by enamel hypoplasia described as “screw-driver” shaped incisors as well as mulberry or moon's molars [207, 306, 307]. Syndromic dental anomalies are dental defects associated with the development of the jaws such as those in OFCs. Van der Woude's syndrome as described before, is also associated with hypodontia [308]. Viral infection with measles, mumps, chickenpox, rubella, and cytomegalovirus *in utero* have been shown to result in enamel hypoplasia and hypocalcification of the teeth in newborns [309]. These defects are mostly observed in the primary dentition. If the infection persists postnatally, enamel defects are observed in the permanent dentition as well [310].

While both viral and bacterial infections have been reported in association with craniofacial defects, few subtle differences do exist in their mechanisms and outcomes. In terms of mechanism, bacterial infections activate the complement pathway and mediate acute inflammatory response. When located intracellularly, they are eliminated by T-cells through the activation of pattern recognition receptors eliciting strong but transient responses. Viral infections often remain subclinical and mediate subtle changes via activation of chronic low-grade inflammation and epigenetic modifications. Viruses can remain latent in host cells and in the carrier commensal pathogens for a long time. Indeed, viral receptors have been found on the fetoplacental unit. Viral infection of the placenta can result in the production of soluble immune factors that could mediate long-term adverse effects in the fetus including developmental defects.

While maternal infections do result in craniofacial defects in the fetus as described above, fortunately, the incidence is rare due to continuous patient monitoring and education. Further studies are needed to understand the effect the pathogens and how they mediate fetal development while maintaining infection at subclinical levels.

## Discussion and Future Directions

Materno-fetal health is a global health concern. Studies in animal models of pregnancy such as the zebrafish, rodents, and large primates, have significantly advanced our understanding of the fetal reprogramming in health and disease, however these are not without limitations. As discussed by Leslie Roberts [311], animal models of pregnancy must be viewed with some skepticism. Soncin et al. reported that a mouse model of pregnancy can only mimic the gene expression patterns of human placenta up to the first 16 weeks [260]. Structurally, the rodent placenta is very different from the human placenta. The exchange of maternal nutrients occurs at the intervillous space in humans, in contrast, in rodents, it happens at the capillary interface. Further, the degree of placentation varies significantly between humans and different animal models [172]. Overall, the choice of animal model is often limited due to ethical and experimental considerations but understanding the outcomes when comparing them to human pregnancy is key. Readers are referred to excellent reviews on the subject of human and non-human primates as study models [312–314].

**Figure 4:**
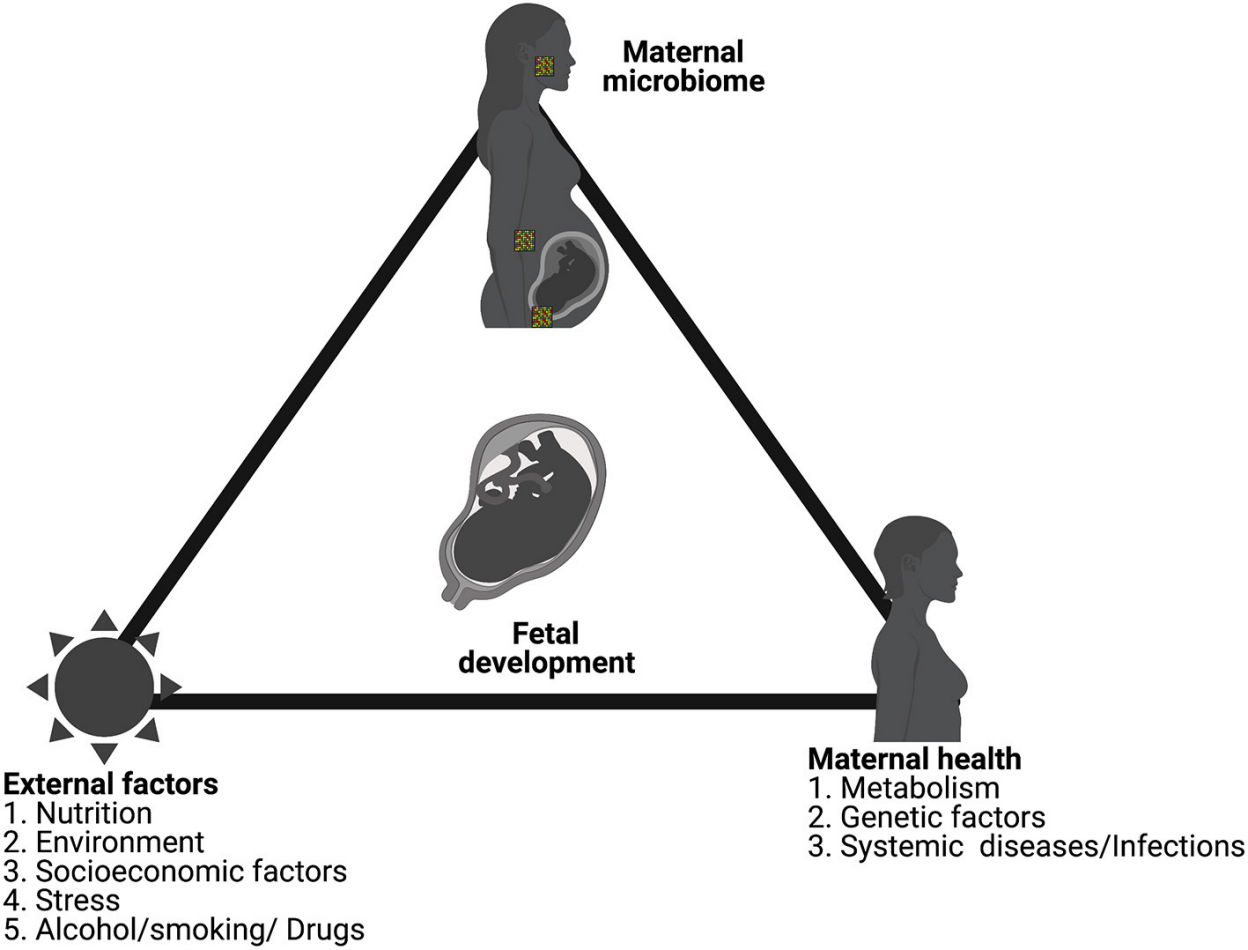
Normal fetal development is a consequence of the overall health of the mother, surrounding environmental factors and the maternal microbiome. Figure created using the BioRender software.

The role of inflammation in adverse outcomes in pregnancy has been well-documented in the literature. While we have discussed inflammation and infection as separate sections in this review, they are deeply interconnected. Though limited, studies have shown isolation of parasites such as plasmodium in the placenta as well as provided evidence for their transplacental transfer [273, 315]. Recently, Humann et al. showed that peptidoglycans from the bacterial cell-wall can cross murine placenta and result in injury to the fetal brain [316]. Reports also highlight the role of microbe derived metabolites in the development of neurodevelopmental disorders [317]. Using germ-free mouse models of pregnancy, Kimura et al. have shown that maternal gut microbiota-derived SCFA can cross the placenta to the developing embryos [318]. Such studies provide experimental support for previous reports linking microbial exposure and fetal gastrointestinal dysfunction as well as the development of neuropsychiatric disorders [317, 319–321]. While the level of sterility of the environment surrounding the fetus is debatable, neither the uterine environment nor the placenta can be considered absolutely sterile. In-fact a distinct microbiome for both have been demonstrated in several studies. As observed with the evolutionary studies in animals, studies in human placenta have suggested mutually learned responses from subclinical infections such as that seen with the insertion of *env*-like *syncytin* gene [322, 323]. As discussed before, several bacteria have been shown to internalize to evade immune responses. Thus, the impact of microbiome and dysbiosis in shaping fetal development is as important as the mother's overall health requires in-depth examination (Figure 4).

Alterations in maternal inflammatory states, microbiome and infections can have long-lasting effects depending on the phase of fetal development. While the evidence regarding the direct transmission of pathogens from mother to fetus remains debated, the passage of pathogen-derived and immune modulator molecules to the fetus is not far-fetched. Pathogen-derived molecules and toxins are small enough to bypass the placental barrier. These remain undetected by the immune system and can induce subclinical responses in both mother and the fetus. If such molecules are capable of inducing epigenetic modifications under laboratory conditions, would it be possible that such modifications could occur in the fetus or result in adverse effects? Maternal immune responses are known to shape fetal development. With the emergence of new pathogens, we will need a deeper understanding of microbiological immune response mechanisms in pregnancy. The influence of the microbiota and their genes (or the microbiome) upon developmental pathways is an emerging field and the available pool of knowledge is limited, providing a convincing argument to support it. Future studies are needed to understand the physiological effects of both symptomatic and subclinical infections in pregnancy and its outcomes and design appropriate biomarkers for their detection.

## Author Contributions

AB, MM, and DA conceptualized the work. AB, MM, VJ, SY, MH, SD, and DA participated in collecting materials and writing sections of the manuscript. All authors read and approved the final manuscript.

## Conflict of Interest

The authors declare that the research was conducted in the absence of any commercial or financial relationships that could be construed as a potential conflict of interest.

## Publisher's Note

All claims expressed in this article are solely those of the authors and do not necessarily represent those of their affiliated organizations, or those of the publisher, the editors and the reviewers. Any product that may be evaluated in this article, or claim that may be made by its manufacturer, is not guaranteed or endorsed by the publisher.
